# Monitoring Autophagy Immunohistochemically and Ultrastructurally during Human Head and Neck Carcinogenesis. Relationship with the DNA Damage Response Pathway †

**DOI:** 10.3390/ijms18091920

**Published:** 2017-09-07

**Authors:** Sophia Havaki, Vassiliki Vlachou, Christos P. Zampetidis, Platonas Selemenakis, Athanassios Kotsinas, Eleni Mavrogonatou, Sophia V. Rizou, Euthymios Kyrodimos, Konstantinos Evangelou, Dimitris Kletsas, Alexandra Giatromanolaki, Vassilis G. Gorgoulis

**Affiliations:** 1 Molecular Carcinogenesis Group, Department of Histology and Embryology, School of Medicine, National and Kapodistrian University of Athens, 75 Mikras Asias Street, 11527 Athens, Greece; shavaki@med.uoa.gr (S.H.); v-vlachou@hotmail.com (V.V.); chzambo@yahoo.gr (C.P.Z.); pselemenakis@gmail.com (P.S.); akotsin@med.uoa.gr (A.K.); sofiriz92@gmail.com (S.V.R.); cnevagel@med.uoa.gr (K.E.); 2 Laboratory of Cell Proliferation and Ageing, Institute of Biosciences and Applications, National Centre for Scientific Research “Demokritos”, 15310 Athens, Greece; elmavro@bio.demokritos.gr (E.M.); dkletsas@bio.demokritos.gr (D.K.); 3 Department of Otolaryngology, Head and Neck Surgery, “Hippokration” General Hospital, National and Kapodistrian University of Athens, 114 Queen Sofia Avenue, 11527 Athens, Greece; ekirodim@med.uoa.gr; 4 Department of Pathology, Democritus University of Thrace, School of Medicine, 68100 Alexandroupolis, Greece; 5 Faculty Institute for Cancer Sciences, Manchester Academic Health Sciences Centre, University of Manchester, Manchester MP13 9PL, UK; 6 Biomedical Research Foundation of the Academy of Athens, 4 Soranou Ephessiou Street, 11527 Athens, Greece

**Keywords:** autophagy, Beclin-1, LC3B, p62, carcinogenesis, DNA damage response

## Abstract

Autophagy is a catabolic process that preserves cellular homeostasis. Its exact role during carcinogenesis is not completely defined. Specifically in head and neck cancer, such information from clinical settings that comprise the whole spectrum of human carcinogenesis is very limited. Towards this direction, we examined the in situ status of the autophagy-related factors, Beclin-1, microtubule-associated protein 1 light chain 3, member B (LC3B) and sequestosome 1/p62 (p62) in clinical material covering all histopathological stages of human head and neck carcinogenesis. This material is unique as each panel of lesions is derived from the same patient and moreover we have previously assessed it for the DNA damage response (DDR) activation status. Since Beclin-1, LC3B and p62 reflect the nucleation, elongation and degradation stages of autophagy, respectively, their combined immunohistochemical (IHC) expression profiles could grossly mirror the autophagic flux. This experimental approach was further corroborated by ultrastructural analysis, applying transmission electron microscopy (TEM). The observed Beclin-1/LC3B/p62 IHC patterns, obtained from serial sections analysis, along with TEM findings are suggestive of a declined authophagic activity in preneoplastic lesions that was restored in full blown cancers. Correlating these findings with DDR status in the same pathological stages are indicative of: (i) an antitumor function of autophagy in support to that of DDR, possibly through energy deprivation in preneoplastic stages, thus preventing incipient cancer cells from evolving; and (ii) a tumor-supporting role in the cancerous stage.

## 1. Introduction

Autophagy is a highly conserved catabolic process, which under physiological conditions contributes to the preservation and restoration of cellular homeostasis through the degradation of damaged intracellular proteins and organelles—the so-called autophagy cargo—in the lysosome [1]. Thus, the functional status of autophagy at basal level warrants the detoxification of the cell and its resistance to nutrient shortage by recycling the breakdown products into metabolic and biosynthetic pathways [2,3]. However, when the intracellular or the extracellular microenvironment is perturbed by chemical, physical or infectious factors that function as stressors, autophagy flux is activated to secure cellular fitness and to restore metabolism and homeostasis. In this respect, dysfunctional autophagic machinery has been associated with numerous diseases, such as neurodegeneration, cardiovascular disorders, infectious conditions, metabolic disorders and cancer [4,5].

In mammals, autophagy is classified into three types depending on the mechanism of cargo delivery to the lysosomes and the involved molecular components: microautophagy [6,7], chaperone-mediated autophagy [8,9] and macroautophagy [10,11]. The latter will be the main topic of the present study. Specifically, macroautophagy (hereafter referred to as autophagy) is controlled by the autophagy-related genes (*ATG*s) and proteins (ATGs). ATGs are responsible for the biogenesis of double-membrane vesicles, called autophagosomes.

**Figure 1:**
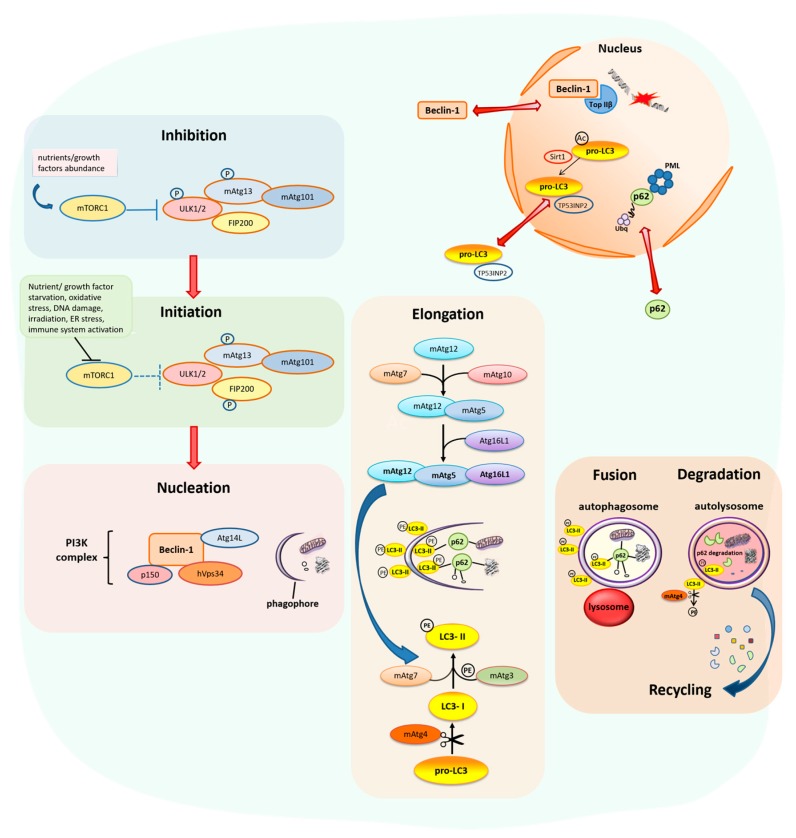
Stages of autophagy. When nutrients and growth factors are abundant, mammalian target of rapamycin complex 1 (mTORC1) associates with and inhibits (T bar, solid line) the ULK1/2-mAtg13-mAtg101-FIP200 complex (ULK complex) by phosphorylation of unc-51-like kinase 1/2 (ULK1/2) and mAtg13. Under starvation and other stress conditions, mTORC1 dissociates (T bar, dotted line) from ULK1/2 leading to its dephosphorylation and allowing it to subsequently phosphorylate focal adhesion kinase family-interacting protein of 200 kDa (FIP200) and mAtg13 triggering (red arrow) the initiation of autophagy. Following initiation, the activated ULK complex participates in the recruitment of additional proteins like Beclin-1, human vacuolar protein sorting-associated protein 34 (hVps34), Atg14-like protein (Atg14L) and p150 that constitute the PI3K complex, which is involved in the nucleation (red arrow) and assembly of an initial double membrane structure, called phagophore, at the site of the autophagic activity. Elongation of the nascent phagophore membrane to form autophagosome is mediated by two ubiquitin-like conjugation systems. In the first system, mAtg12 is covalently bound to mAtg5 assisted by the mAtg7 and mAtg10 enzymes. Subsequently, Atg16L1 associates with the mAtg12-mAtg5 conjugate forming a mAtg12-mAtg5-Atg16L1 complex. The latter promotes (blue arrow) the function of the second system which involves LC3. The initial form of LC3 (pro-LC3) is cleaved by the protease mAtg4 to produce the active cytosolic form LC3-I. LC3-I is then activated by mAtg7 and covalently conjugated to phosphatidylethanolamine (PE) by mAtg3 to form LC3-II, which then associates with the elongated autophagophore and resides in its inner and outer membrane. As autophagosome maturation proceeds p62 identifies and delivers ubiquitinated, misfolded and aggregated proteins or damaged organelles into the autophagosome via its ubiquitin-associated and LC3-binding domains. The mature, closed autophagosome is then fused with lysosomes to form autolysosome. The engulfed cargo into autolysosome, along with p62 and LC3-II, is exposed to the lysosomal hydrolases that degrade it and then degradation products are transferred into the cytosol for further recycling via anabolic procedures. In addition to their cytoplasmic presence, Beclin-1, LC3B and p62 autophagy markers are found in the nucleus through nucleocytoplasmic shuttling (double red arrows), carrying out autophagy and non-autophagy related functions.

Autophagy is a multistep process characterized by the initiation, nucleation, elongation, fusion and degradation stages [7,12] as shown in Figure 1. Among the key regulatory factors of autophagy are Beclin-1, the microtubule-associated protein 1 light chain 3 (LC3) and p62. Specifically, Beclin-1 is a member of the class III phosphatidylinositol 3-kinase (PI3K) complex, acting as a platform for the assembly of the components that constitute it and stimulate its activity. Moreover, Beclin-1 regulates the nucleation and assembly of the initial double membrane structures at the sites of autophagic activity, which consist the autophagosome precursors, known as phagophores [12,13,14]. Subsequently, elongation of the growing phagophores’ membrane is driven by two processes; first, by the covalent linkage of mAtg5 to mAtg12 and then to Atg16-like 1 protein (Atg16L1) resulting in the production of the mAtg12-mAtg5-Atg16L1 complex that in turn promotes the second process by conjugating phosphatidylethanolamine (PE) to the active cytosolic form of LC3, called LC3-I. The latter derives from the pro-LC3 after its cleavage via the protease mAtg4. PE-conjugated LC3-I (lipidated), known as LC3-II, is then capable to integrate into the phagophores’ membrane and decorate it, residing in both the inner and outer side of the membrane [15,16]. As autophagosome maturation proceeds, p62, the best characterized autophagy substrate and adaptor, identifies and delivers selectively ubiquitinated, misfolded and aggregated proteins or damaged organelles into the autophagosomes through an ubiquitin-associated and a LC3-binding domains [17,18]. Mature autophagosomes can then fuse directly with lysosomes, forming autolysosomes, for the degradation of the autophagy cargo after its exposure to acidic hydrolases in the interior of lysosomes, thus completing the process of the autophagy flux. p62 is exclusively degraded through autophagy. Therefore, it is considered to be an established marker for monitoring the autophagy flux determining the actual ability for intracellular components’ degradation [19]. Degradation products are released into the cytoplasm for recycling. In addition, LC3-II located at the cytosolic surface of the autolysosome undergoes mAtg4-mediated decoupling from PE, while intra-autophagosomal LC3-II is also degraded [20].

**Figure 2:**
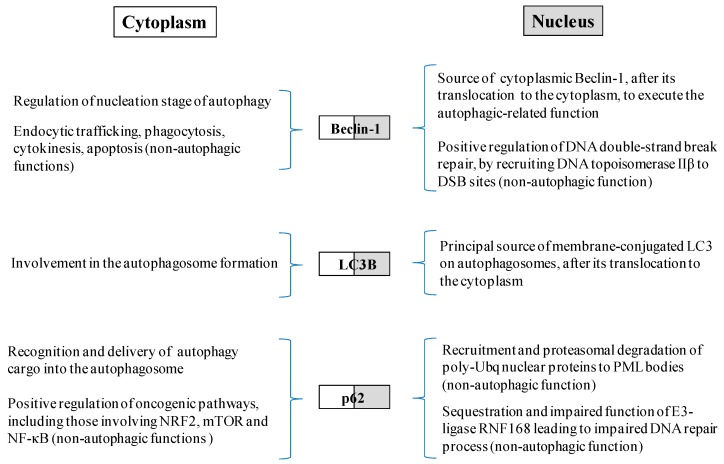
Schematic representation of the cytoplasmic and nuclear functions of the autophagy—related factors Beclin-1, LC3B and p62.

Although Beclin-1, LC3 and p62 are considered to be principally cytosolic proteins, they are also localized in the nucleus [20,21,22] (Figure 1 and Figure 2). In both cellular compartments these factors have been shown to exert autophagy, as well as non-autophagy related functions [23,24,25,26,27]. Specifically, the presence of Beclin-1 in the nucleus and its transport to the cytoplasm is tightly linked to its autophagic function and tumor-suppressive role. Prerequisite for the nucleocytoplasmic shuttling of Beclin-1 is the presence of an intact and functional nuclear export signal (NES) [22]. Moreover, it has been recently shown that the nuclear localization of Beclin-1 in stressed cells with functional autophagy is associated with the regulation of DNA double-strand break (DSB) repair, irrespectively of its autophagic function, by interacting with DNA topoisomerase IIβ and by recruiting it to DSB sites, ensuring genomic integrity [27]. On the other hand, the nuclear pool of LC3 serves as a principal source of membrane-conjugated LC3 on autophagosomes, after its translocation to the cytoplasm. Particularly, starvation-induced autophagy leads to LC3 deacetylation by sirtuin 1 (SIRT1) deacetylase in the nucleus and to the subsequent binding of LC3 with the tumor protein 53 inducible nuclear protein 2 (TP53INP2) promoting its translocation from the nucleus to the cytoplasm [28,29]. Furthermore, p62 shuttles between nucleus and cytoplasm rapidly and constantly, with preferential distribution in the cytoplasm. In the nucleus, p62 contributes to the recruitment of polyubiquitinated nuclear proteins or protein aggregates to promyelocytic leukemia bodies (PML) and is involved in their proteasomal degradation in this cellular compartment [21]. Moreover, under conditions of autophagy inhibition, localization of p62 in the nucleus, leads to sequestration and impaired function of the E3-ligase ring-finger protein 168 (RNF168), an upstream component of the DNA damage response (DDR) pathway, resulting in reduced recruitment of DNA repair proteins at the DSB sites [30,31].

Although under normal conditions autophagy has a homeostatic function and constitutes a cytoprotective mechanism against tumorigenesis, in cancer autophagy has been found to play either a tumor-suppressive or a tumor-promoting role in a cancer-type and -stage dependent context [32,33,34]. Many studies, based on in vitro and in vivo model systems, have shown that during cancer initiation, autophagy can act as a tumor suppressor by removing damaged proteins and organelles and by reducing free radicals and oxidative stress which may lead to genomic instability and tumorigenesis [34,35]. On the other hand, in advanced stages of carcinogenesis it appears to exert a tumor-promoting role, possibly representing a key element of an adaptation program that cancer cells develop to deal with adverse microenvironmental conditions, such as hypoxia, nutrient deprivation and high metabolic demands due to increased growth and proliferation rates [33,36]. Although the role of autophagy in various carcinomas has been extensively studied, its role in the progression of head and neck cancer is not clearly defined [37,38]. Given that head and neck cancer is becoming a leading cause of death worldwide [37], elucidating the role of autophagy in the development of this type of cancer may highlight potential beneficial therapeutic modalities.

According to the oncogene-induced DNA damage model for cancer development we have proposed, oncogene activation disrupts normal DNA replication from the earliest stages of cancer development (precancerous stages), leading to replication stress, triggering the DDR pathway, promoting genomic instability and cancer progression [39,40,41,42,43]. In brief, the DDR is a network of cellular pathways that sense, signal and repair DNA lesions. The DNA damage is recognized by sensor proteins that recruit and activate the transducer kinases. The latter, assisted by mediators, relay the signal to the upstream effector kinases, which activate the downstream effectors. These in turn recruit the appropriate DNA repair pathway in response to the specific type of DNA lesion. Depending on the complexity of the DNA lesion each DNA repair pathway may act independently or in cooperation with other repair mechanisms. Activation of the DNA damage signaling checkpoints provides the necessary time for repair as they inhibit the cyclin–dependent kinase (CDK) complexes to slow down or arrest the cell cycle progression. If the damage is extensive or not effectively repaired, the cell undergoes apoptosis, senescence, or acquires chromosomal aberrations, which may lead to genomic instability and carcinogenesis [44,45,46]. Given that the DDR is a highly demanding energy process and is activated from the earliest stages of carcinogenesis [39,40,42,47,48], a key question that emerges is whether autophagy, that acts as back-up energy reserve under stress conditions [49], follows a similar activation pattern in head and neck carcinogenesis.

To address this question, we monitored the immunohistochemical patterns of Beclin-1, LC3B and p62 followed by ultrastructural analysis in clinical settings of head and neck cancers that cover the whole histopathological spectrum of cancer development (normal, precancerous and cancerous stages) in each patient. The fact that every clinical panel was derived from the same patient renders it unique, as it lacks the disadvantages of heterogeneous sources that may influence the “biological readings” of the final outcome. Importantly, the activation status of the DDR pathway in this clinical setting has already been thoroughly examined [39,41,48], allowing the direct correlation with the aforementioned autophagy markers.

## 2. Results and Discussion

### 2.1. Defining the Immunohistochemical Profiles of Beclin-1, LC3B and p62 in Human Head and Neck Prencancerous and Cancerous Lesions

**Figure 3:**
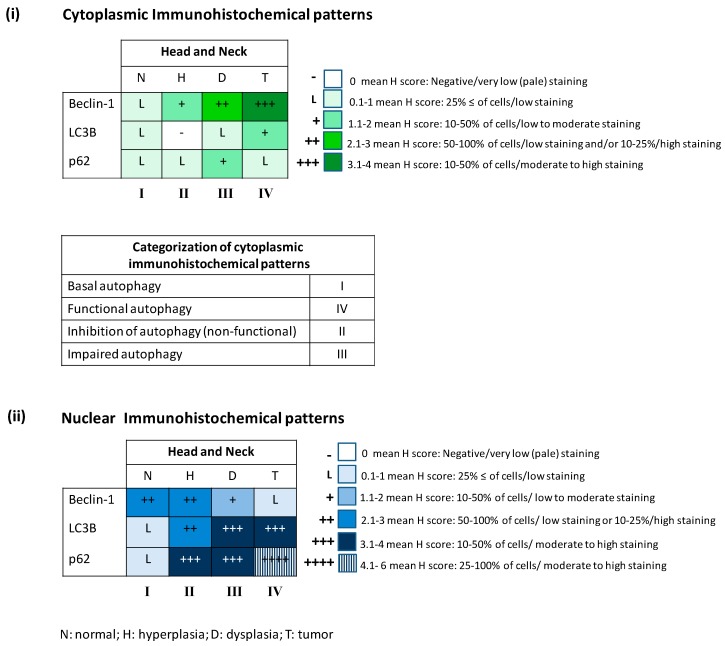
Color-coded charts showing (**i**) the cytoplasmic and (**ii**) the nuclear immunohistochemical patterns of the three studied autophagy-related markers (Beclin-1, LC3B and p62) in head and neck lesions. The color-coding is based on the classification of mHscore values as described in the Materials and Methods section. Four cytoplasmic and nuclear immunohistochemical patterns resulted, respectively, from the combination of the color-coded classes for each autophagy marker in every stage of cancer. Based on autophagic activity, the cytoplasmic patterns were grouped into four categories. The first category refers to basal autophagy represented by pattern I and characterized by low immunostaining of Beclin-1, LC3B and p62. The second category refers to competent autophagic activity represented by pattern IV, and was characterized by a lower p62 immunohistochemical staining compared to LC3B, indicative of proficient ability of intracellular components to be degraded via autophagy. The third category refers to inhibition of autophagy represented by pattern II, characterized by a faint staining of LC3B, since this is a key autophagy-related factor for autophagosome formation. The fourth category refers to impaired autophagic activity represented by pattern III. This was characterized by an increased p62 immunohistochemical staining compared to LC3B, indicative of a cytoplasmic p62 accumulation possibly resulting in the disturbance of cell function and homeostasis.

The examined material, although unique from a clinical perspective, it is archival (formalin fixed and paraffin embedded) and can be evaluated only by immunohistochemistry and electron microscopy. However, a detailed view of the expression profile and in situ subcellular localization (cytoplasmic and nuclear) of Beclin-1, LC3B and p62 can be provided since all stages of carcinogenesis can be observed in the same section from the same patient. The latter minimizes interlesion immunohistochemical staining variability. Most importantly, immunohistochemical evaluation of these factors was performed in serial sections from each lesion. This approach has the advantage of providing a “snapshot” of the whole molecular cascade at a particular location of the lesion and at a specific time-point during carcinogenesis. Given that Beclin-1, LC3B and p62 reflect in general the nucleation, elongation and degradation stages of autophagy respectively (Figure 1) [7,12], their immunohistochemical evaluation, corroborated by ultrastructural analysis, could mirror a gross estimation of the autophagic flux. Considering the multistep sequel and dynamic inter-correlation of these molecules during the autophagy process [7,12,50], we developed and applied a semiquantitative evaluation algorithm (Figure 3, Table 1, see also corresponding section in Materials and Methods).

Based on it, we defined as reference immunohistochemical pattern the following: cytoplasmic Beclin-1 (Low)—cytoplasmic LC3B (Low)—cytoplasmic p62 (Low). This profile was referred from now on as: Cyt-Beclin-1(L)/LC3B(L)/p62(L). Of note, cytoplasmic LC3B expression levels refer to the punctate immunohistochemical pattern (Figure S1), which is correlated with the autophagosome-specific LC3B-II form [15]. In addition, p62 levels are estimated relatively to the LC3 expression status, since low cytoplasmic p62 expression in comparison to LC3 denotes functional integrity of autophagy [26]. The reference pattern was deduced from the corresponding “normal” tissue, adjacent to the pathological ones, sharing with them the same stressful microenvironment. Given that nuclear localization of the triad Beclin-1/LC3B/p62 has also been related to normal autophagy function (Figure 2) [21,22,28,29], the pattern Nuc-Beclin-1(++)/LC3B(L)/p62(L) was incorporated to the above mentioned reference profile, and was deduced also from the normal areas (Figure 3 and Table 1). Specifically, nuclear LC3B (Nuc-LC3B) should also be considered as a physiological feature of a functional autophagic flux since it serves as the principle source of membrane-conjugated LC3 on autophagosomes [29]. Similarly, transport of nuclear Beclin-1 (Nuc-Beclin-1) to the cytoplasm is related to functional autophagy and tumor suppression and depends on the integrity of its NES [22]. Furthermore, as stated earlier, nuclear p62 (Nuc-p62) normally participates in the recruitment of polyubiquitinated nuclear proteins or protein aggregates to PML bodies for proteasomal degradation [21]. All other expression patterns of the triad Beclin-1/LC3B/p62, cytoplasmic or nuclear, were regarded abnormal (Figure 3 and Table 1), most likely reflecting dysfunctional autophagic flux or implying non-autophagic activity, such as that of nuclear Beclin-1 regulating DSB repair via topoisomerase-IIβ under conditions of cellular stress [27] (see also Introduction).

### 2.2. Evaluating Immunohistochemically and Ultrastructurally Autophagy in Human Head and Neck Carcinogenesis

**Figure 4:**
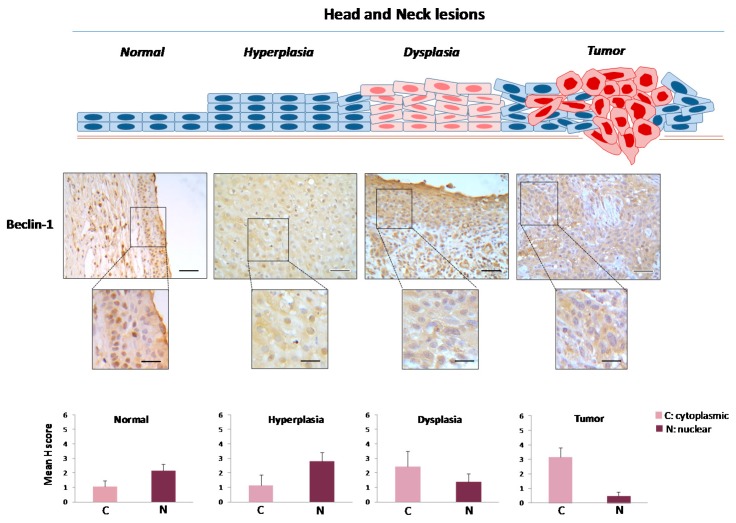
Immunohistochemical expression patterns of Beclin-1 in head and neck lesions. Representative images of cytoplasmic and nuclear immunohistochemical localization of Beclin-1 in normal laryngeal epithelium, hyperplastic and dysplastic lesions, as well as in tumor areas from the same patient. Bars in the histograms denote the cytoplasmic and nuclear mHscores of Beclin-1 in each stage (± SE: standard error). Blue cells in two lines depict normal laryngeal epithelium; blue cells in four lines depict hyperplastic epithelium; pink cells depict dysplastic epithelium; pink cells with red nuclei depict cancer cells. Scale bars: 100 μm; scale bars of insets: 50 μm.

**Figure 5:**
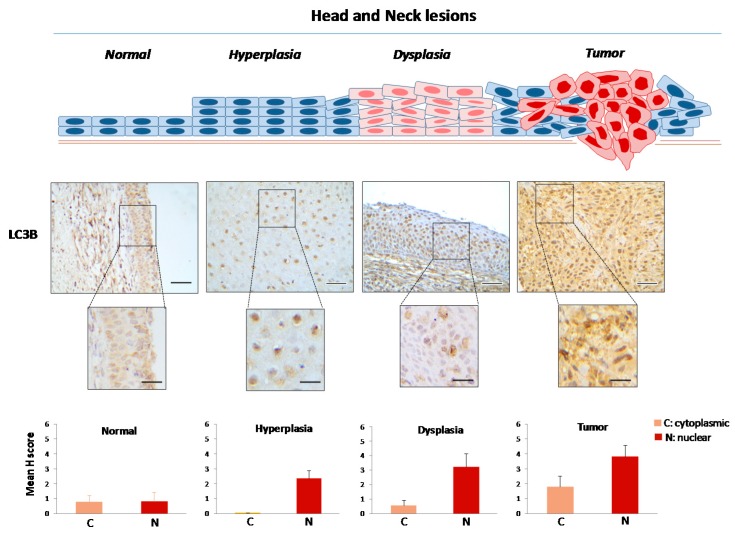
Immunohistochemical expression patterns of LC3B in head and neck lesions. Representative images of cytoplasmic and nuclear immunohistochemical localization of LC3B in normal laryngeal epithelium, hyperplastic and dysplastic lesions, as well as in tumor areas from the same patient. Bars in the histograms denote the cytoplasmic and nuclear mHscores of LC3B in each stage (± SE: standard error). The cytoplasmic mHscores refer to the punctate localization pattern of LC3B (see also Figure S1) representing the LC3B-II lapidated form that participates in the autophagosome formation. Scale bars: 100 μm; scale bars of insets: 50 μm.

**Figure 6:**
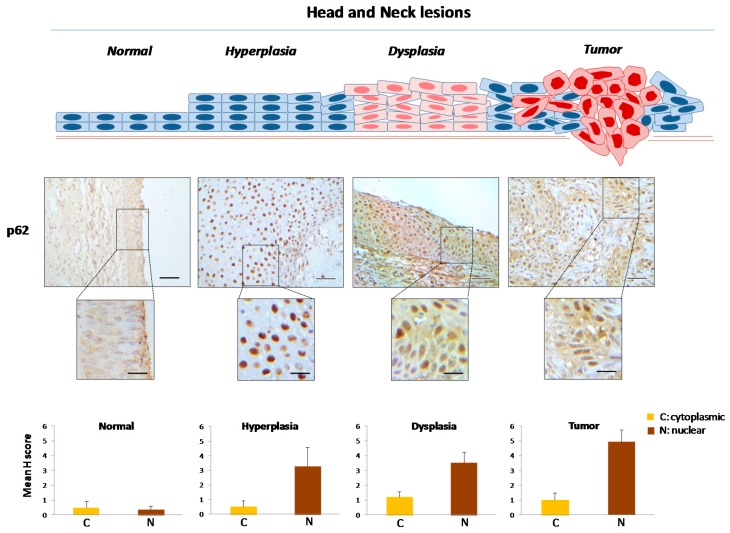
Immunohistochemical expression patterns of p62 in head and neck lesions. Representative images of cytoplasmic and nuclear immunohistochemical localization of p62 in normal laryngeal epithelium, hyperplastic and dysplastic lesions, as well as in tumor areas from the same patient. Bars in the histograms denote the cytoplasmic and nuclear mHscores of p62 in each stage (± SE: standard error). Scale bars: 100 μm; scale bars of insets: 50 μm.

Normal laryngeal epithelium exhibited a [Cyt-Beclin-1(L)/LC3B(L)/p62(L)]/[Nuc-Beclin-1(++)/LC3B(L)/p62(L)] profile (reference pattern indicating basal level autophagy) (Table 1; Figure 3, Figure 4, Figure 5 and Figure 6 and Figure S1), while hyperplastic and dysplastic lesions displayed the abnormal patterns [Cyt-Beclin-1(+)/LC3B(−)/p62(L)]/[Nuc-Beclin-1(++)/LC3B(++)/p62(+++)] and [Cyt-Beclin-1(++)/LC3B(L)/p62(+)]/[Nuc-Beclin-1(+)/LC3B(+++)/p62(+++)] respectively (Table 1; Figure 3, Figure 4, Figure 5 and Figure 6 and Figure S1), suggestive of an impaired autophagy process in these preneoplastic stages. Particularly, the increased cytoplasmic p62 levels observed in both types of premalignant lesions may contribute to oncogenic transformation, given that p62 positively regulates many oncogenic pathways, including those involving nuclear factor erythroid 2-related factor (NRF2), mammalian target of rapamycin (mTOR) and nuclear factor-κB (NF-κΒ), in the context of non-autophagic functions [26,51]. Additionally, hyperplastic lesions were characterized by a strikingly declined autophagy, based on the faint staining of LC3B, as it is a key autophagy-related factor for autophagosome formation. Given that autophagy serves as a back-up energy source under stress conditions, such as hypoxia and nutrient starvation [49,52] favoring survival, the decreased autophagy-related pattern in hyperplastic lesions implies an energy deficit state that could prevent incipient cancer cells from evolving. If the reduction of energy load is severe and rapid, below a critical limit it is likely that cell death (apoptosis) will be triggered [53,54], via DDR as previously shown [48]. However, restoration of autophagic competence in cells that manage to avoid apoptosis will facilitate their progressive evolution from dysplastic to full blown cancer cells.

Carcinomas demonstrated a [Cyt-Beclin-1(+++)/LC3B(+)/p62(L)]/[Nuc-Beclin-1(L)/LC3B(+++)/p62(++++)] profile (Table 1; Figure 3, Figure 4, Figure 5 and Figure 6 and Figure S1) indicating a restored, potentially functional autophagy, given the increased expression of the cytoplasmic Beclin-1 and LC3B and the relatively lower p62 levels.

**Figure 7:**
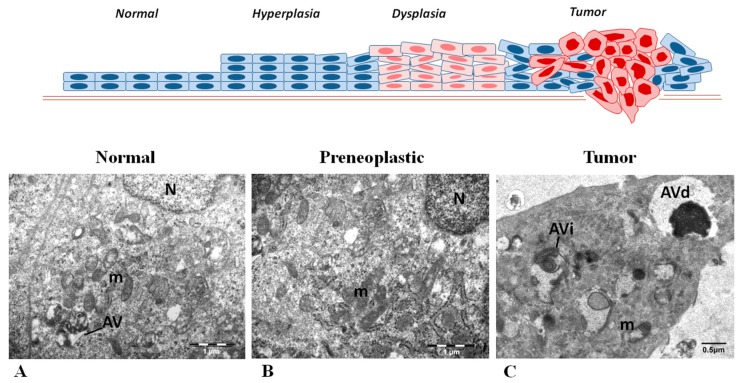
Representative electron micrographs of normal epithelial (**A**), preneoplastic (**B**) and tumor (**C**) laryngeal cells from the same patient. In normal laryngeal epithelium, few autophagic vacuoles (AV) were observed (**A**). In preneoplastic (dysplastic) lesions the autophagic vacuoles were rarely present (**B**). In contrast, tumor cells exhibited increased numbers of autophagic vacuoles that were either initial (AVi) or degradative (AVd) ones (**C**). Quantitative analysis of autophagic vacuoles showed the following values: 4.8 ± 0.5 in normal cells, 1.3 ± 0.6 in preneoplastic cells and 12.4 ± 0.8 in cancer cells. m, mitochondrion; N, nucleus. Scale Bars: A and B: 1 μm; C: 0.5 μm.

All the above immunohistochemical findings and suggestions were clearly supported by the observations of the ultrastructural analysis of the same cases (Figure 7). Particularly, in the cytoplasm of normal laryngeal epithelial cells, few autophagic vacuoles (AV) were observed indicating autophagic activity at basal level (Figure 7A). In preneoplastic cells the AV were hardly present (Figure 7B) signifying the limited autophagosome formation in these lesions. In contrast, an increased number of AV were observed in tumor cells (Figure 7C) and at different stages of the autophagic process, i.e., initial and degradative autophagic vacuoles, without the predominance of neither type, pointing to an activated but efficient autophagy in head and neck cancer. Otherwise, in case of impaired autophagy process, accumulation of AV at initial or degradative stage would be observed. In support to the observed increased autophagy in the cancerous stage, conducting a nutrient starvation experiment in the HN13 HNSCC cell line that mimics the adverse nutrient microenvironment of carcinomas, we noticed an enhancement of autophagy, indicating its prosurvival role (Figure S2).

Notably, nuclear Beclin-1 showed decreasing expression from normal to cancerous state, while p62 had a reverse expression profile (Figure 4 and Figure 6, respectively). These results are consistent with a previous study [55], which demonstrated that high cytoplasmic expression of Beclin-1 accompanied by low nuclear levels is associated with tumor progression in oral cancer. Furthermore, high cytoplasmic expression of Beclin-1 has been associated with tumor aggressiveness in nasopharyngeal carcinoma [56] and has been related to unfavorable prognosis and aggressive clinical features in oral squamous cell carcinomas [57,58]. In contrast, high expression of Beclin-1 has been considered a favorable prognostic factor for laryngeal squamous cell carcinomas, by others [59]. Further investigation is needed to clarify this controversy. Moreover, given the non-autophagic role of nuclear Beclin-1 in DNA repair [27], the observed gradual decrease of Beclin-1 in the nucleus in the successive stages of laryngeal carcinogenesis, may possibly reflect an impaired DNA repair process that could fuel genomic instability.

Since p62 has been shown to contribute to the proteasomal degradation of nuclear proteins at the PML bodies [21], the excessive nuclear levels of p62 in preneoplastic and malignant lesions may lead to aberrant proteasomal targeting of DDR and DNA repair components, thus promoting genomic instability and cancerous progression. Within this vein, it has been reported that p62 participates in the proteasomal degradation of filamin A and Rad51 recombinase, essentials factors of homologous recombination, following irradiation [60]. In addition, we have recently shown that reduction of Rad51 elicits a switch from high-fidelity homologous recombination to a lower-fidelity repair process mediated by Rad52 resulting in genomic instability and cancer progression [61]. Moreover, p62 is known to suppress the RNF168-mediated chromatin ubiquitination at the sites of DNA damage resulting in the reduced recruitment of DNA repair proteins [30]. Thus, along with the effect of decreased nuclear Beclin-1 levels, and in accordance with the oncogene-induced DNA damage model for cancer development, a breach of the error-free DNA repair machinery will take place further fueling genomic instability [42].

### 2.3. Relationship between Autophagy and the DDR Pathway during Human Head and Neck Cancer Development

**Figure 8:**
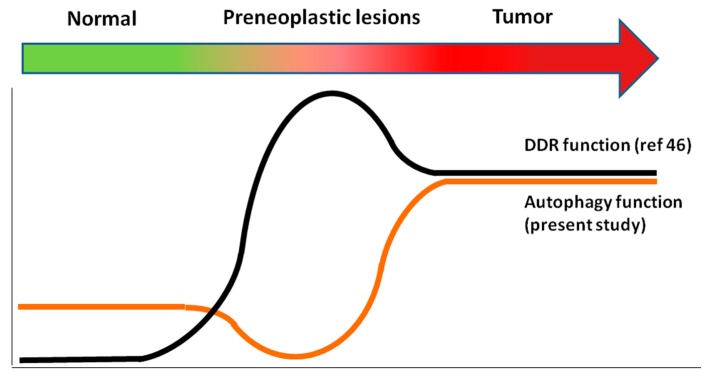
Schematic representation of the interrelationship of DDR pathway and autophagy during carcinogenesis (for details see results-discussion and conclusion).

In the same head and neck clinical samples, the signaling cascade of the DDR pathway has been extensively studied and shown to be activated from hyperplasia to full-blown cancer [48]. Correlating these data with the results obtained in this study, two association patterns were defined (Figure 8). Specifically, in the precancerous stages there was an inverse relationship between autophagy and the DDR pathway, whereas in the tumors they correlated showing a parallel track (Figure 8).

## 3. Material and Methods

### 3.1. Human Samples

Human head and neck samples consisted of formalin-fixed, paraffin-embedded laryngeal squamous cell carcinomas (*n* = 19) specimens, from surgically resected lesions. Paraffin blocks were obtained from the Laboratory of Histology and Embryology, School of Medicine, National and Kapodistrian University of Athens. All samples were collected according to the local ethical committee guidelines (approval no: 5758/03-2003). Patients, who had undergone chemo-, radio- or immunotherapy prior to surgical resection, were excluded to avoid any interference with the expression profile of the examined factors.

All human specimens included in the same slide normal tissue and the entire spectrum of carcinogenesis of each particular malignancy, rendering this material unique. Specifically, in the cases of laryngeal squamous lesions from each patient, normal epithelium (squamous and respiratory), hyperplastic and dysplastic epithelium, as well as invasive squamous cell carcinoma were assessed. All samples used have been described previously [41,48].

### 3.2. Immunohistochemistry (IHC)

Method: An immunoperoxidase staining was performed on 2.5 μm serial paraffin sections of the human biopsies using the Ultravision LP Detection System kit from Thermo Scientific. Heat-induced antigen retrieval was carried out in citrate buffer pH 6.00 for 25 min. Antibodies used were polyclonal anti-BECN1 (1/200, Santa Cruz Biotechnology, Code No: sc-11427), monoclonal anti-LC3B (1/50, clone 5F10, Nanotools, Code No: 0231-100/LC3-5F10) and monoclonal anti-SQSTM1/p62 (1/200, Abcam, Code No: ab56416). Notably, the monoclonal antibody of clone 5F10 is specific to LC3B protein [16] and particularly to both LC3B forms I and II. All samples were incubated with the primary antibodies overnight at 4 °C. For color development we used 3,3′-diaminobenzidine tetrahydrochloride (DAB) and hematoxylin, as counterstain.

Evaluation of immunohistochemistry: Immunohistochemical reaction for each antibody was evaluated by two independent qualified pathologists (VV, KE), using a Leica DM LB2 optical microscope and counting 10 high-power fields (×400) per slide and for each lesion (normal epithelium, hyperplastic lesions, dysplastic lesions and carcinomas), separately. Intraobserver variability was minimal (*p* < 0.05). Evaluation of cytoplasmic and nuclear staining of the three autophagy markers (Beclin-1, LC3B and p62) was taken into consideration. Regarding the evaluation of the cytoplasmic staining of LC3B, only the punctate pattern was taken into consideration (Figure S1), since it is associated with the autophagosome-specific LC3B-II isoform. The diffuse cytoplasmic staining pattern corresponds to the LC3B-I isoform which is not related to autophagosome formation. The method of histological score (Hscore) assignment has been previously described [62,63]. Immunohistochemical staining was evaluated on the basis of the immunostaining intensity and the proportion of stained cells. Specifically, staining intensity was classified into 4 grades: 0 (pale or no staining), 1 (low staining), 2 (moderate staining) and 3 (high staining). The percentage of positively stained tumor cells was scored in 4 grades: 0 (0–10%), 1 (10–25%), 2 (25–50%) and 3 (50–100%). Mean Hscores (mHscores) were calculated as follows:

(1) $$\frac{\left(\mathrm{Intensity}\;\mathrm{reader}\;1\times \mathrm{Percentage}\;\mathrm{reader}\;1\right)+(\mathrm{Intensity}\;\mathrm{reader}\;2\times \mathrm{Percentage}\;\mathrm{reader}\;2)}{2}$$

#### Selected Fields Were Identical in all Serial Sections to Enable Optimal Comparison

Obtained values of mHscores were categorized in five classes for cytoplasmic immunostaining depicted with green shading (Figure 3i) and six classes for nuclear immunostaining depicted with blue shading (Figure 3ii). The highest range of each class was determined by the value obtained after the multiplication of the grade of stained cell proportion by the grade of immunostaining intensity, hence by the values 0, 1, 2, 3, 4, 6. The lowest range of each class was determined by the highest value of the previous class, as shown in Figure 3i,ii. Consequently, the class of mHscore value 0 represents negative or minimal immunostaining; the class of mHscore values 0.1–1 represents low immunohistochemical staining in <25% of counted cells; the class of mHscore values 1.1–2 represents low to medium immunohistochemical staining in 10–50% of counted cells; the class of mHscore values 2.1–3 represents low immunohistochemical staining in the majority of the cells (50–100%) and/or high staining in 10–25% of counted cells; the class of mHscore values 3.1–4 represents medium to high immunohistochemical staining in 10–50% of counted cells and the class of mHscore values 4.1–6 represents medium to high immunohistochemical staining in the majority of the cells (25–100%). This latter class was not included in cytoplasmic immunostaining, because the corresponding mHscore values were not obtained. For presentation purposes of immunohistochemical patterns in the text, each class was symbolized as follows: (−) for class of mHscore value 0, (L) for class of mHscore values 0.1–1, (+) for class of mHscore values 1.1–2, (++) for class of mHscore values 2.1–3, (+++) for class of mHscore values 3.1–4 and (++++) for class of mHscore values 4.1–6 (Figure 3 and Table 1). mHscore values for each studied tissue are presented in Supplemental Materials (file: mHscore S1).

Controls: Paraffin sections of human placenta were used as positive controls for the markers Beclin-1 and p62, while human kidney paraffin sections were used as positive controls for LC3B, as suggested by the antibody manufacturers (Figure S3). Tissue paraffin sections stained by omitting application of the primary antibody served as negative controls.

### 3.3. Transmission Electron Microscopy (TEM)

For the ultrastructural confirmation of the presence of autophagic vacuoles, tissue fragments from head and neck lesions were fixed in 2.5% glutaraldehyde made up in phosphate buffer saline (PBS) 0.01 M, pH 7.4 for 1h at room temperature and postfixed in 1% aqueous OsO_4_ for 30 min at 4 °C. They were then dehydraded in graded series of ethyl alcohol, followed by propylene oxide (PO) treatment, infiltrated gradually in a mixture of Epon/Araldite resins diluted in PO and finally embedded in fresh epoxy resin mixture. Ultrathin (80–90 nm thick) sections were cut with a Diatome diamond knife on a Leica Ultracut R ultramicrotome and were collected onto 200 mesh uncoated copper grids. Ultrathin sections were observed with a FEI Morgagni 268 transmission electron microscope and micrographs were taken with an Olympus digital camera.

## 4. Conclusions and Further Questions

In conclusion, our findings suggest that autophagy has a dual role during head and neck carcinogenesis. In preneoplastic stages, the inverse relationship between autophagy and the DDR pathway could be viewed as a potential concerted action aiming to destroy incipient cancer cells, possibly through energy deprivation. On the other hand, in the cancerous stages restoration of autophagy and its parallel action with the DDR pathway, suggests a supportive role for tumor development. In accordance is the fact that, despite improvements in treatment strategies in head and neck cancer, tumor cells frequently develop resistance to chemotherapeutic drugs due to the adaptive autophagy induction, which plays a prosurvival role. For this reason, inhibition of autophagy is often chosen as adjuvant therapy to sensitize head and neck cancer cells to chemotherapy; thus increasing cell death potential [38,64,65]. However, although paradoxical, initial activation of autophagy followed by its abrupt inhibition could prove to be an alternative beneficial chemosensitization strategy, as it has been recently shown [66]. Specifically, combining a first line chemotherapeutic agent with an autophagy inducer and an autophagy inhibitor in cancer cell lines further enhances cell death. This triplet drug treatment exerted antioncogenic properties in a PI3K/Akt/mTOR pathway-dependent manner. Sustained autophagy activity followed by autophagy inhibition proved to be lethal for cancer cells [66]. Ischemia-reperfusion injury [67] constitutes a clinical analogue of such a process.

Whether these findings are shared also by other types of cancer remains to be clarified. A question to be addressed is the functional interplay and mechanistic features of the autophagy and DDR association during the various phases of head and neck carcinogenesis. Another issue is to clarify the implication of senescence in this interaction, as suggested by certain studies [47]. Given that a revolutionary compound has been generated for the evaluation of senescent cells in various biological materials, including archival ones [68], this method could prove to be an invaluable tool in this quest. Moreover, answers to these questions could pinpoint novel putative therapeutic targets.

## Figures and Tables

**Table 1:** (**i**) Cytoplasmic and (**ii**) nuclear immunohistochemical patterns of the triad Beclin-1/LC3B/p62 in each stage of head and neck cancer.

(i) Cytoplasmic Immunohistochemical Patterns					(ii) Nuclear Immunohistochemical Patterns				
Autopahgy-Related Factors	Head and Neck				Autopahgy-Related Factors	Head and Neck			
	N	H	D	T		N	H	D	T
Beclin-1	L	+	++	+++	Beclin-1	++	++	+	L
LC3B	L	−	L	+	LC3B	L	++	+++	+++
p62	L	L	+	L	p62	L	+++	+++	++++

N: normal; H: hyperplasia; D: dysplasia; T: tumor; (−) for class of mHscore value 0; (L) for class of mHscore values 0.1–1; (+) for class of mHscore values 1.1–2; (++) for class of mHscore values 2.1–3; (+++) for class of mHscore values 3.1–4; (++++) for class of mHscore values 4.1–6.

## Supplementary Materials

Supplementary materials can be found at www.mdpi.com/1422-0067/18/9/1920/s1.

## Abbreviations

ATG	Autophagy-related gene
Atg16L1	Atg16-like 1 protein
Atg14L	Atg14-like protein
CDK	Cyclin–dependent kinase
DDR	DNA damage response
DSB	DNA double-strand break
FIP200	Focal adhesion kinase family-interacting protein of 200 kDa
hVps34	Human vacuolar protein sorting-associated protein 34
IHC	Immunohistochemistry
LC3	Microtubule-associated protein 1 light chain 3
mTOR	Mammalian target of rapamycin
mTORC1	Mammalian target of rapamycin complex 1
NES	Nuclear export signal
NRF2	Nuclear factor erythroid 2-related factor
NF-κΒ	Nuclear factor-κB
PE	Phosphatidylethanolamine
PI3K	Phosphatidylinositol 3-kinase
PML	Promyelocytic leukemia bodies
RNF168	Ring-finger protein 168
SIRT1	Sirtuin 1
SQSTMQ/p62	Sequestosome 1/p62
TP53INP2	Tumor protein 53 inducible nuclear protein 2
ULK1/2	Unc-51-like kinase 1/2
