# Supplementary material for: Monitoring Autophagy Immunohistochemically and Ultrastructurally during Human Head and Neck Carcinogenesis. Relationship with the DNA Damage Response Pathway
Source: Int J Mol Sci. 2017 Sep 7;18(9):1920. doi: 10.3390/ijms18091920 (PMC5618569; doi:10.3390/ijms18091920)
Supplement: Supplementary file 1 [file ijms-18-01920-s001.zip › ijms-221566 SI.pdf]

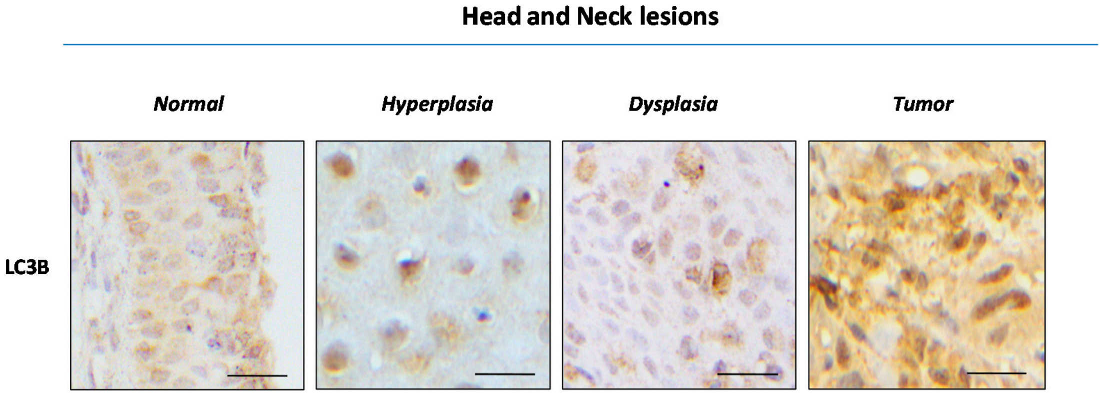

Figure S1. Higher magnification of the insets presented in **Figure 5** depicting the punctate pattern of cytoplasmic positivity of LC3B. Scale Bars: 0.5 µm.

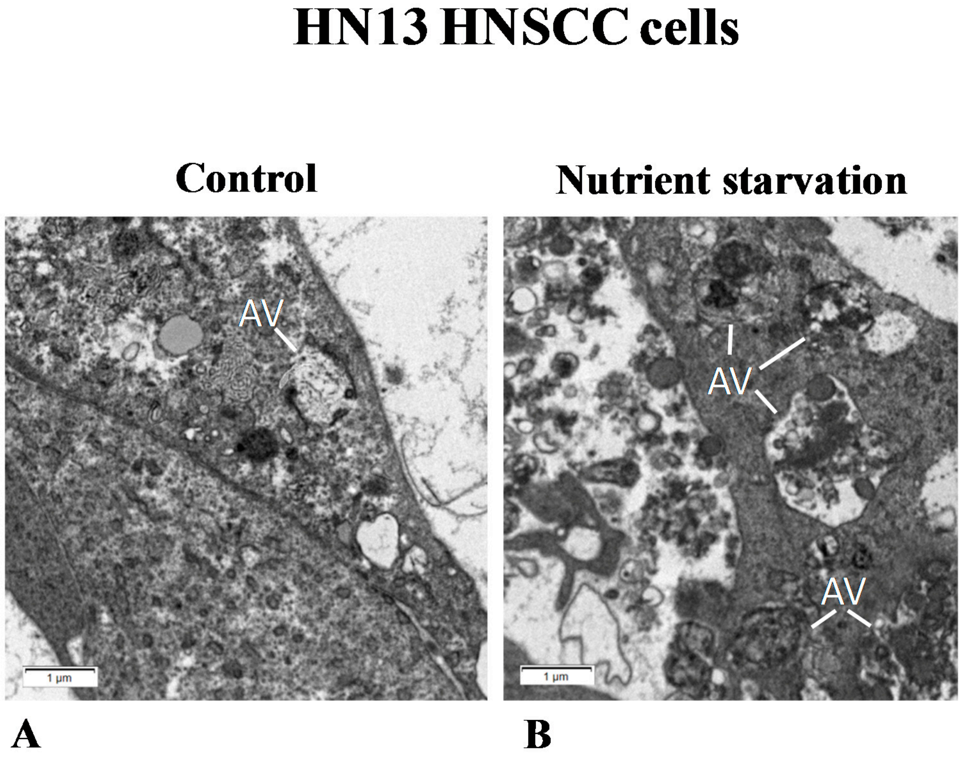

Figure S2. Electron micrographs of HN13 HNSCC cell line after nutrient starvation treatment (B), showing an increase of autophagic vacuoles (AV) compared to the control cells (A). Scale Bar: 1µm.

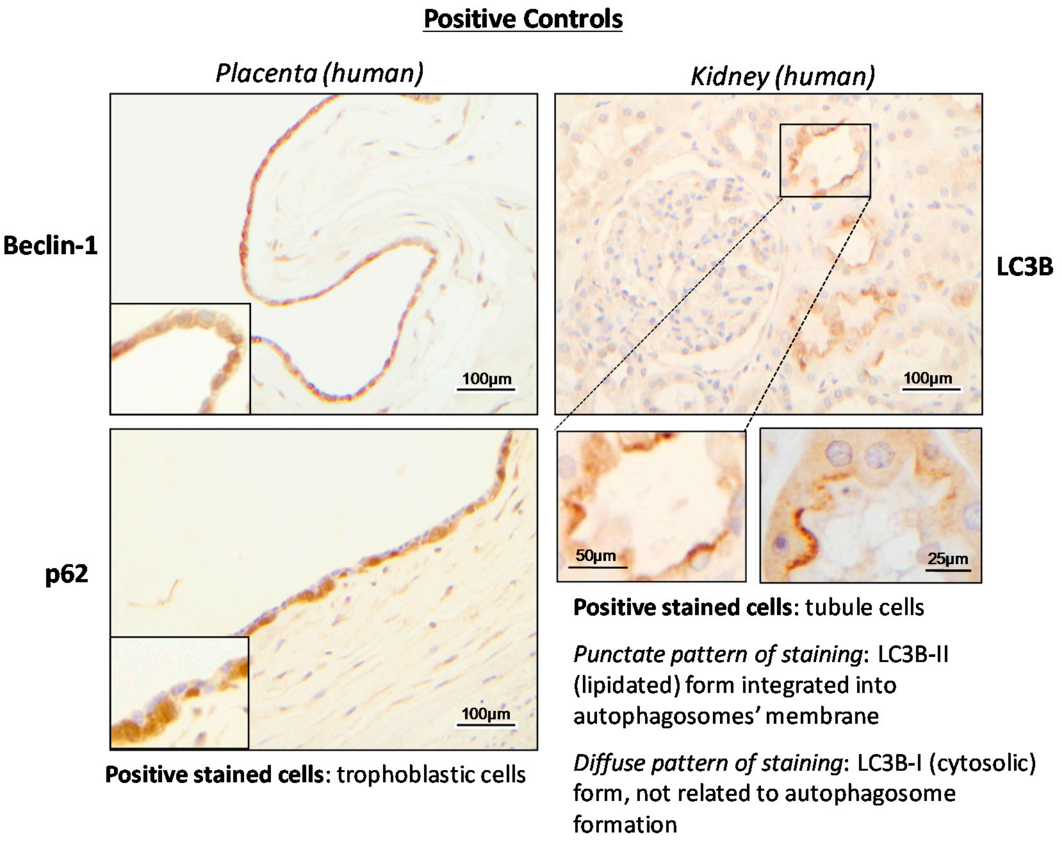

Figure S3. Positive control immunostaining reactions for the markers BECN1 (Beclin-1) and SQSTTM1/p62 in paraffin sections of human placenta, and for LC3B in human kidney paraffin sections, validating the specificity of the employed antibodies as suggested by the antibody manufacturers.

Supplementary Method: HN13 HNSCC cell line was cultured as previously described [SR1]. Nutrient starvation experiment conducted according to the protocol described by Zhu and colleagues, 2017 [SR2]. Cells were treated for Electron Microscopy analysis according to the procedure that has been described in the 3.3 section of the main text.

Supplemental Reference

SR1. Younis, R.H.; Han, K.L.; Webb, TJ. Human head and heck squamous cell carcinoma–associated semaphorin 4D induces expansion of myeloid-derived suppressor cells. *J Immunol.* **2016**, *196* (3): 1419- 1429. DOI: 10.4049/jimmunol.1501293.

SR2. Zhu, W.; Qu, H,; Xu, K.; Jia, B.; Li, H.; Du, Y.; Liu, G.; Wei, H-J.; Zhao, H-Y. Differences in the starvation-induced autophagy response in MDA-MB-231 and MCF-7 breast cancer cells. *Animal Cells and Systems*, **2017**, *21*,190 – 198.
