# Supplementary material for: Monitoring Autophagy Immunohistochemically and Ultrastructurally during Human Head and Neck Carcinogenesis. Relationship with the DNA Damage Response Pathway
Source: Int J Mol Sci. 2017 Sep 7;18(9):1920. doi: 10.3390/ijms18091920 (PMC5618569; doi:10.3390/ijms18091920)
Supplement: Supplementary file 1 [file ijms-18-01920-s001.zip › ijms-221566 Suppl Data_ mHscores.pdf]

Monitoring autophagy immunohistochemically and ultrastructurally during human head and neck carcinogenesis. Relationship with the DNA Damage Response pathway.

mHscores values

Head and Neck lesions

		Cytoplasmic	Sterror	Nuclear	Sterror
**Beclin1**	Normal	1,00	0,44	2,17	0,44
	Hyperplasia	1,15	0,71	2,80	0,63
	Dysplasia	2,43	1,06	1,40	0,54
	Tumor	3,14	0,65	0,46	0,30

		Cytoplasmic	Sterror	Nuclear	Sterror
**LC3B**	Normal	0,79	0,42	0,82	0,60
	Hyperplasia	0,02	0,02	2,36	0,53
	Dysplasia	0,54	0,38	3,22	0,92
	Tumor	1,80	0,73	3,80	0,80

		Cytoplasmic	Sterror	Nuclear	Sterror
**p62**	Normal	0,47	0,45	0,36	0,24
	Hyperplasia	0,52	0,43	3,29	1,30
	Dysplasia	1,20	0,37	3,51	0,75
	Tumor	1,02	0,46	4,93	0,82
